# Non-communicable diseases related multimorbidity, catastrophic health expenditure, and associated factors in Ernakulam district

**DOI:** 10.3389/fpubh.2024.1448343

**Published:** 2024-12-04

**Authors:** Sarah Joy, Aswathy Sreedevi, Renjitha Bhaskaran

**Affiliations:** ^1^Department of Community Medicine, Amrita Institute of Medical Sciences Amrita Vishwa Vidyapeetham, Kochi, Kerala, India; ^2^Department of Biostatistics, Amrita Institute of Medical Sciences Amrita Vishwa Vidyapeetham, Kochi, Kerala, India

**Keywords:** non-communicable diseases, multimorbidity, health expenditure, catastrophic, Kerala

## Abstract

**Background:**

Multimorbidity, the coexistence of two or more chronic conditions in an individual, has emerged as a significant public health challenge with profound economic implications, exerting substantial strain on healthcare systems and economies worldwide. This study aimed to estimate the prevalence of non-communicable diseases (NCD) related multimorbidity, catastrophic health expenditure (CHE), and associated factors among adults aged ≥40 years in Ernakulam district.

**Methods:**

A community-based cross-sectional study was conducted among 420 individuals aged ≥40 years using population probability sampling. The tools used were the Multimorbidity Assessment Questionnaire for Primary Care (MAQ-PC), the WHO STEPS Questionnaire, and the Patient Health Questionnaire-9 (PHQ-9), which assessed non-communicable diseases, diet, physical activity, smoking, alcohol consumption, and depression. A pretested semi-structured questionnaire collected data on health and household expenditures. Multimorbidity was defined as having two or more self-reported NCDs, and CHE was identified when health costs exceeded 10% of a household’s expenditure in the past month. Multivariable logistic regression was performed to find independent predictors of multimorbidity and CHE.

**Results:**

The mean age of participants was 60.02 ± 10 years. The prevalence of NCD multimorbidity was 42.6% (95% CI: 37.9–47.3%). The most common dyad was diabetes and hypertension (24.5, 95% CI: 20.4–28.6%). Higher odds of multimorbidity were observed in those aged ≥60 years (aOR = 3.03, 95% CI: 1.95–4.73), unmarried/widowed/divorced (aOR = 2.15, 95% CI: 1.28–3.63), unemployed (aOR = 1.81, 95% CI: 1.14–2.87), and tobacco users (aOR = 3.72, 95% CI: 1.85–7.48). Approximately 32.4% (95% CI: 25.5–39.3%) of households incurred catastrophic health expenditure (CHE) by treating adults with multimorbidity. Age ≥ 60 (aOR = 2.39, 95% CI: 1.99–5.77) and use of outpatient services (aOR = 4.09, 95% CI: 2.01–8.32) were independently associated with higher odds of CHE. IP services and each additional morbidity add ₹22,082.37 (*β* = 0.557, *p* < 0.001, 95% CI: ₹17,139.88– ₹27,024.86) and ₹1,278.75 (*β* = 0.128, *p* = 0.044, 95%CI: ₹35.58–₹2,521.92) to healthcare costs, respectively.

**Conclusion:**

The high prevalence of multimorbidity and associated CHE among individuals over 60 years highlights the urgent need for the National Programme for the Prevention and Control of Non-Communicable Diseases to prioritise multimorbidity and its management, especially above 60 years within this age group.

## 1 Introduction

Multimorbidity presents a significant challenge to health services, both currently and in the foreseeable future (1). It refers to the simultaneous presence of two or more chronic conditions within the same individual. Although they are frequently associated with non-communicable diseases (NCDs), these problems are not just related to them (2). The rising older population and the escalating incidence of chronic illnesses underscore multimorbidity as a critical issue in global public health. Up to 90% of people worldwide, including various age groups, suffer from multimorbidity (3). The prevalence of multimorbidity in South Asia varies widely, ranging from 4.5 to 83% (4). In India, a 2017 study reported an overall prevalence of 23.3%, with Kerala showing the highest prevalence at 42% (5). Another community-based study from the South Indian state of Andhra Pradesh estimated multimorbidity at 58.5% (6), while research across seven Indian states found a prevalence of 30.7% (7). A community-based study in Kerala reported a prevalence of 45.4% (8), whereas two hospital-based studies showed varying prevalence rates, from 16.2 to 39.8% (9, 10).

Understanding the factors contributing to multimorbidity is essential for devising successful strategies aimed at the early identification of at-risk individuals and preventing future health complications (11). These factors encompass a wide array of influences, including individual characteristics as well as broader societal and environmental factors. Given India’s present demographics, it is more important to identify multimorbidity in all adult groups, not only the older population (12). Even though multimorbidity is known to be significantly correlated with age, additional research into potential risk factors is required.

Kerala is in an advanced stage of epidemiological transition characterised by the rising burden of diabetes and hypertension, with multimorbidity emerging as a significant public health concern impacting approximately half of the adult population within the productive age group (8). While several studies have attempted to estimate the prevalence of multimorbidity, the majority have focussed on primary healthcare settings and the older population.

Multimorbidity poses significant health challenges, leading to adverse outcomes such as disability, increased mortality rates, poor quality of life, frequent hospitalisations, heightened utilisation of medical resources, and escalated healthcare expenditure (13). Many health systems, geographic locations, illness combinations, and individual characteristics such as age and social disadvantage will likely have very diverse effects on the costs and resources of healthcare due to multimorbidity. In some instances, the treatment of individuals with multimorbidity can impose a significant financial burden on families, leading to Catastrophic Health Expenditure (CHE). The incidence of CHE is on the rise, often driven by high out-of-pocket healthcare spending (14). Additional factors contributing to CHE include lack of health insurance, poverty, the type of medical conditions, and the absence of a robust government-funded healthcare system.

The objective of universal health coverage, as outlined in Target 3.8 of the United Nations’ Sustainable Development Goals for 2030, is to establish a healthcare system wherein individuals can access services without facing financial hardships (15). On the contrary, according to an analysis of Social Consumption Health data from the National Sample Survey Organisation’s 75th round which was carried out in India between 2017 and 2018, 46.7% of households incurred catastrophic healthcare costs (using a 10% threshold) as a result of any non-communicable disease (NCD). This percentage increased to approximately 63.3% in the presence of multimorbidity, compared to approximately 39.4% for any communicable disease (16).

Therefore, a comprehensive understanding of the epidemiology of multimorbidity, particularly related to non-communicable diseases (NCDs), is essential for restructuring healthcare services to offer integrated care for individuals with multiple chronic conditions. There is a notable knowledge gap that exists on the community-level prevalence of multimorbidity among the adult population, its factors, and the diverse patterns of multimorbidity. The catastrophic health expenditure encountered by households in the treatment of these conditions has also not been studied much.

The current study aimed at determining the prevalence of NCD-related multimorbidity at the community level, along with its associated factors among adults aged 40 years and above in Ernakulam district, Kerala. We also attempted to estimate the proportion of households that encountered catastrophic health expenditure by treating such adults.

## 2 Materials and methods

### 2.1 Study participants

A community-based cross-sectional study was conducted in rural and urban areas of Ernakulam district, situated in the central part of Kerala in Southern India, which has a population of 3.2 million. A cluster sampling with probability proportionate to size sampling (PPS) was carried out, and 20 clusters from the district were chosen for the study. A sampling frame with all the Community Development (CD) Blocks/towns of Ernakulam district was initially prepared, and the population of all the areas was listed. The total population was divided by the number of clusters to determine the sampling interval, and a random number was generated with the help of an online random number generator. A panchayat/block was then selected as the first cluster containing the cumulative population less than or equal to the random number. The sampling interval was then added 19 times to obtain the 20 clusters. From each cluster, a ward was identified randomly using computer-generated random numbers.

Adults aged 40 years and above who had been residing in Ernakulam district were included in the study. The exclusion criteria were those who had been staying in the district for less than 6 months. The sample size was determined based on a study by Rohini et al. (8), which reported a prevalence of 45.4% for non-communicable disease multimorbidity. With an absolute precision of 7 and applying a design effect of 2, the sample size was calculated to be 388, and we have included 420 participants in our study.

### 2.2 Study variables and definitions

The outcome variables in the study were NCD multimorbidity and households that incurred catastrophic health expenditure (CHE). ‘Multimorbidity’ is defined as the coexistence of two or more chronic conditions in the same individual (17) and a household is said to incur ‘Catastrophic Health Expenditure’, when the total expenditure on health exceeds 10% of total household expenditure or income in the preceding month (SDG 3.8.2) (18). Data on NCD multimorbidity were collected by identifying the coexistence of two or more chronic conditions over the past 12 months, as self-reported by the study participants and verified against their corresponding medical records. To calculate the health expenditure, details regarding outpatient services, inpatient services, home care services, and other monthly healthcare charges in the previous month were gathered. Outpatient services’ expenses consisted of consultation fees, cost of medicines, investigations, and transportation costs. Inpatient services in the previous month details were gathered regarding the amount of total hospital bill, cost of medicines and investigations (not included in the main hospital bill), cost of food and transport, the duration of hospital stay, and the caregiver’s or the accompanying person’s income loss. Inquiries were made regarding the fees associated with home care services, including charges for a home nurse, consultation at home, and the expenses related to medications or investigations after a visit by a healthcare provider. The independent variables collected included sociodemographic details, information regarding diet, physical activity, smoking and alcohol consumption, anthropometric measurements, health insurance patterns, and monthly household expenditure. To calculate monthly household expenditure, we included house rent, loan repayment, travel, food, media, educational expenditure, electricity and water charges, and domestic help.

### 2.3 Study tools

Sociodemographic details were collected by using a structured questionnaire. Data on NCD multimorbidity were collected using the Multimorbidity Assessment Questionnaire for Primary Care (MAQ-PC) developed and validated in India (19) and translated into the local language (Malayalam). The Patient Health Questionnaire-9 (PHQ-9) (20) was used to screen and assess the severity of depression in individuals. Each question was scored on a scale from 0 to 3, representing “Not at all,” “Several days,” “More than half the days,” and “Nearly every day,” and PHQ-9 scores of 5, 10, 15, and 20 represented mild, moderate, moderately severe, and severe depression, respectively. Information on diet, physical activity, smoking, and alcohol consumption was collected using the WHO Stepwise Approach to Surveillance (STEPS) Questionnaire (21). A pretested semi-structured questionnaire was used to gather data relating to health expenditure and household spending incurred by individuals in the preceding month.

### 2.4 Statistics

The data collected using Epicollect 5 software (22) were exported to Microsoft Excel, and the analysis was conducted using SPSS version 21. The quantitative variables were expressed as mean and SD, and qualitative variables were mentioned as proportions. The chi-square test was used to assess the factors associated with multimorbidity and catastrophic health expenditure. All determinants with a *p*-value in chi-square test <0.2 were further used in the logistic regression modelling, using a backward conditional model. A *p*-value of less than 0.05 was considered to be statistically significant. The multivariable logistic regression helped to determine the independent predictors of multimorbidity and catastrophic health expenditure and was expressed as an odds ratio with a 95% confidence interval. Multiple linear regression analysis was conducted to determine the independent predictors influencing OOPE. Latent class analysis (LCA) was performed to identify subgroups (latent classes) based on patterns of multimorbidity. The optimal number of latent classes was determined using model fit indices such as the Akaike Information Criterion (AIC), Bayesian Information Criterion (BIC), deviance statistic (G^2^), and chi-square goodness of fit (χ^2^) (23, 24). Class population proportions and probabilities of non-communicable diseases (NCDs) within each class were then reported. Analysis for this study was conducted using the ‘poLCA’ package in RStudio V.1.1.463 (R Studio).

### 2.5 Ethical considerations

Prior to the commencement of the study, an ethical clearance certificate (ECASM-AIMS-2023-006) was obtained from the Amrita School of Medicine Ethical Committee. Informed consent was taken from all the study participants.

## 3 Results

The mean age of the study participants was 60.02 ± 10 years, and more than a third 151 (36%) of the participants were 60–69 years old. The majority (278, 66.2%) of the participants were females. The majority (296, 70.5%) had an education above high school, and only 18 (4.3%) had no formal schooling. Only 47 participants (11.2%) reported being smokers, and only 45 (10.7%) reported alcohol consumption. Approximately half of the participants 196 (46.8%) were in the obese category (Table 1).

**Table 1:** General characteristics of the study population (*n* = 420).

Sl. No	Characteristics	Category	Frequency	Percentage
	Mean age (SD)	60.02 ± 10 years		
1	Age (years)	40–49	73	17.4
		50–59	118	28.1
		60–69	151	36.0
		≥70 years	78	18.6
2	Sex	Male	142	33.8
		Female	278	66.2
3.	Area of residence	Rural	245	58.3
		Urban	175	41.7
4	Religion	Hindu	182	43.3
		Christian	166	39.5
		Muslim	72	17.1
5	Marital status	Currently married	327	77.9
		Widowed	88	21.0
		Others (unmarried, divorced)	5	1.1
6	Education	No formal schooling	18	4.3
		Primary school	49	11.7
		Secondary school	57	13.6
		High school and above	296	70.5
7	Occupation	Homemaker	226	53.8
		Currently employed	141	33.6
		Unemployed	53	12.6
8	Type of family	Nuclear	212	50.5
		Three generation	204	48.6
		Joint family	4	1
9	Family size	≤ 4	212	50.5
		>4	208	49.5
10	Socioeconomic status	APL	276	65.7
		BPL	144	34.3
11	Tobacco use	Ever used	47	11.2
		Never used	373	88.8
12	Alcohol consumption	Ever used	45	10.7
		Never used	375	89.3
13	Fruit intake	<4 days/week	251	59.8
		≥4 days/week	169	40.2
14	Vegetable intake	<4 days/week	17	4.0
		≥4 days/week	403	96.0
15	Physical activity (*n* = 116)	<600 MET min/week	44	37.9
		≥600 MET min/week	72	62.1
16	BMI	Underweight (<18.5)	8	1.9
		Normal (18.5–22.9)	104	24.8
		Overweight (23–24.9)	112	26.5
		Obese (≥ 25)	196	46.8

APL, above poverty line; BPL, below poverty line; BMI, body mass index.

**Figure 1:**
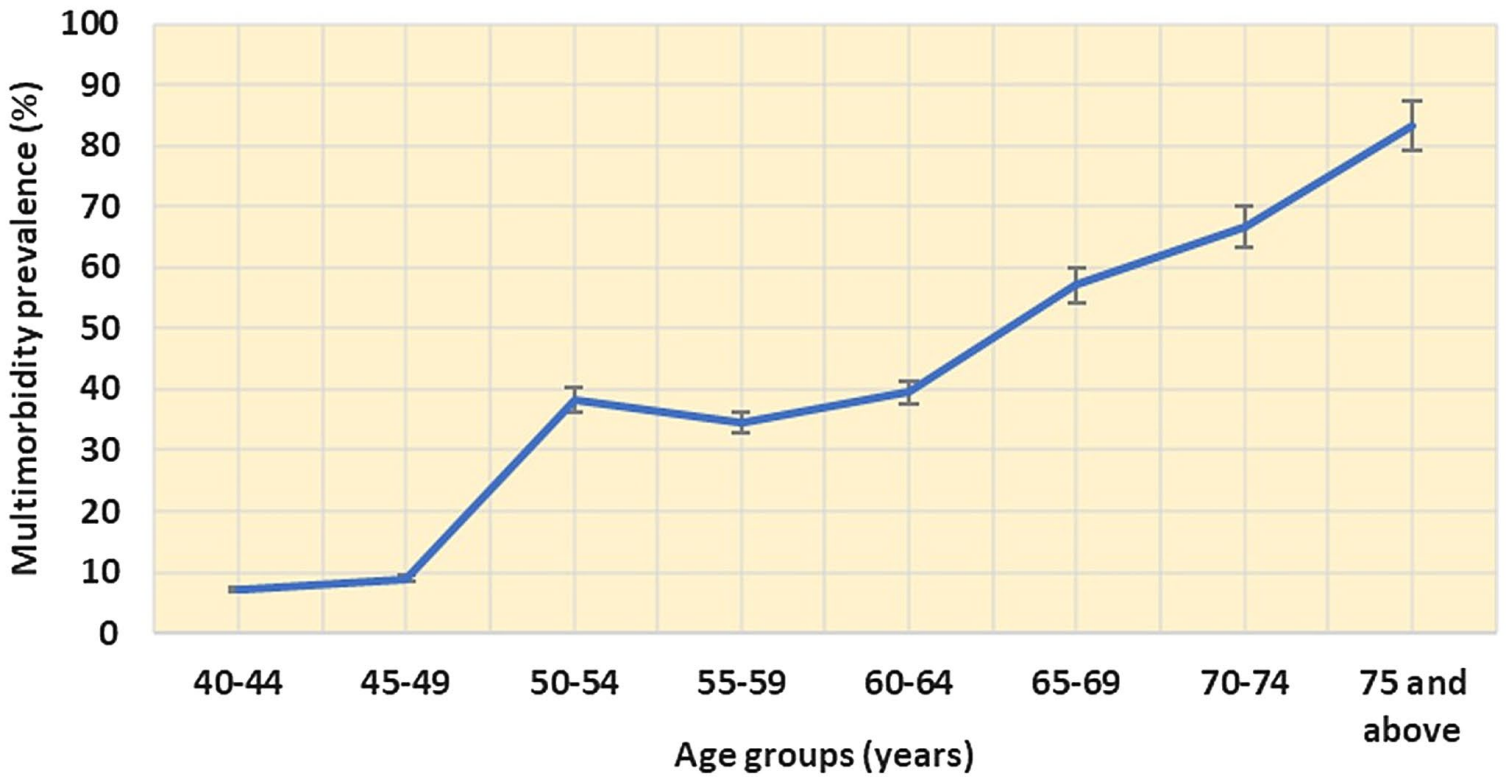
Distribution of multimorbidity based on age groups.

The overall prevalence of NCD multimorbidity was found to be 42.6% (95% CI: 37.9–47.3) and less than a third (31.7%) of the study participants reported only one condition. The prevalence of multimorbidity begins to noticeably increase from the age of 45 (8.9, 95% CI: 6.2–11.6) and continues to rise steadily until the age of 75, reaching 83.3% (95% CI: 81.5–85.1; Figure 1). Approximately a quarter of the study population 21.9% (95% CI: 17.9–25.0) reported having two chronic conditions (dyad). Additionally, 13.3% (95% CI: 10.0–16.6), 3.3% (95% CI: 1.6–5.0), and 4% (95% CI: 2.1–5.9) reported having three (triad), four (quad), and more than five (penta and above) chronic conditions, respectively.

**Figure 2:**
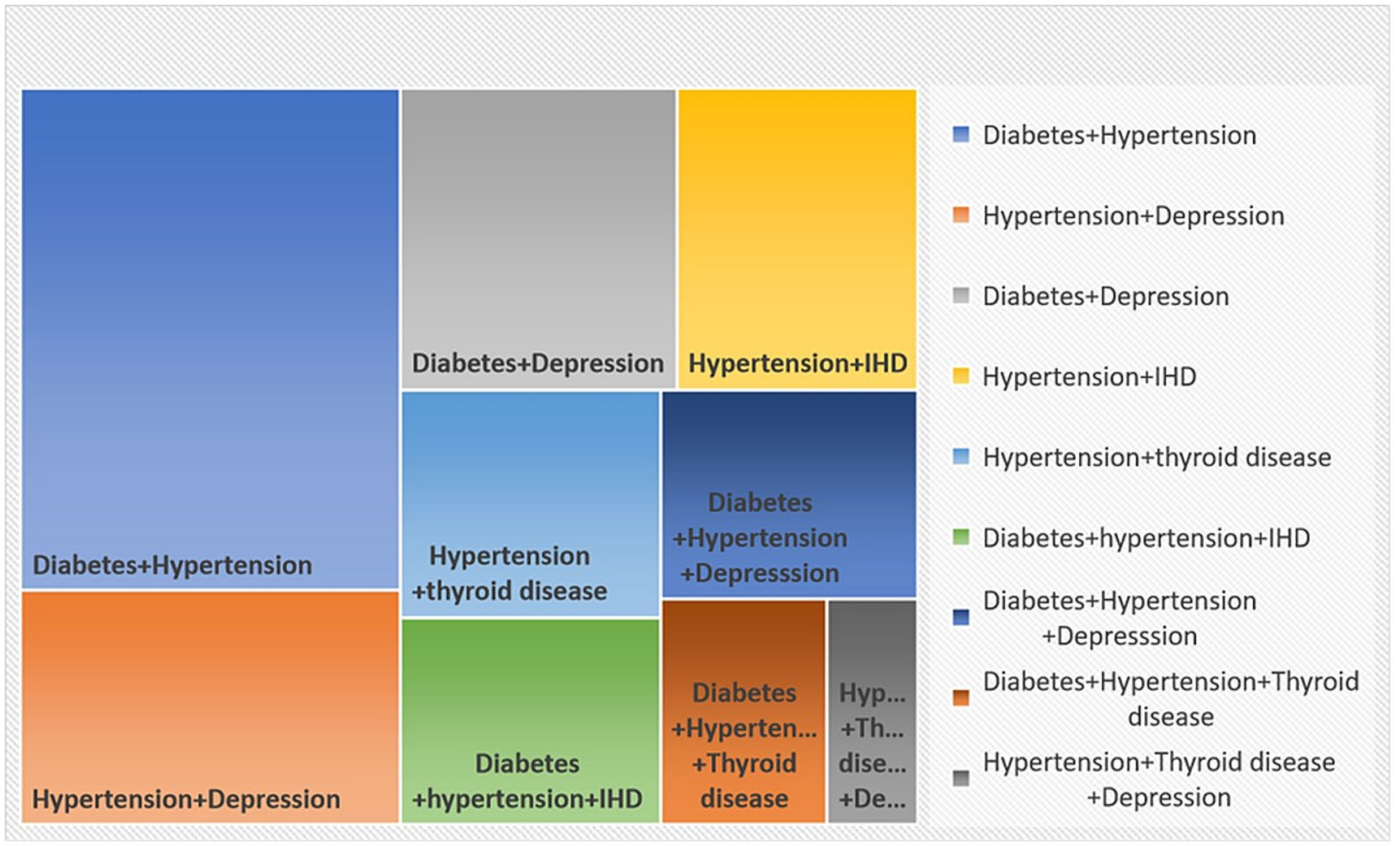
Distribution of multimorbidity patterns using treemap.

The study identified diabetes and hypertension as the most common dyad (pairs of conditions) observed in multimorbidity 24.5% (95% CI: 20.4–28.6; Figure 2). The second most common pair was hypertension–depression (11.4, 95% CI: 8.4–14.4) followed by diabetes and depression (10.7, 95% CI: 7.7–13.6).

Based on the bivariate analysis to determine the association of multimorbidity and its determinants, factors such as age, marital status, education, occupation, type of family, family size, and tobacco use were significant with a *p*-value less than 0.05 (Table 2).

**Table 2:** Multimorbidity and its determinants.

Sl. No	Socio-demographic characteristics	Total (n)	MM present n (%)	Crude OR (with 95% CI)	*p*-value
1	Age in years				
	≥ 60 years	229	130 (56.8)	3.80 (2.50–5.77) Ref	<0.001*
	< 60 years	191	49 (25.7)		
2	Sex				
	Female	278	113 (40.6)	0.78 (0.52–1.18) Ref	0.253
	Male	142	66 (46.5)		
3	Area of residence				
	Rural	245	100 (40.8)	0.83 (0.56–1.24) Ref	0.377
	Urban	175	79 (45.1)		
4	Religion				182
	Hindu	182	73 (40.1)	0.84 (0.48–1.48) 0.60 (0.34–1.07) Ref	0.149
	Christian	166	80 (48.2)		
	Muslim	72	26 (36.1)		
5	Marital status				
	Others (unmarried, widowed, divorced)	93	57 (61.3)	2.66 (1.65–4.27) Ref	<0.001*
	Currently married	327	122 (37.3)		
6	Education				
	Below high school	124	73 (58.9)	2.56 (1.67–3.94) Ref	<0.001*
	High school and above	296	106 (35.8)		
7	Occupation				
	Unemployed	279	131 (47.0)	1.71 (1.12–2.61) Ref	0.012*
	Currently employed	141	48 (34.0)		
8	Type of family				
	Nuclear	212	74 (34.9)	0.52 (0.35–0.77) Ref	0.001*
	Others	208	105 (50.5)		
9	Family size				
	≤ 4	212	94 (37.5)	0.59 (0.39–0.87) Ref	0.009*
	>4	208	85 (50.3)		
10	Socio-economic status				
	APL	276	109 (39.5)	0.69 (0.46–1.03) Ref	0.073
	BPL	144	70 (48.6)		
11	Tobacco use				
	Ever used	47	32 (68.1)	3.28 (1.71–6.26) Ref	< 0.001*
	Never used	373	147 (39.4)		
12	Alcohol consumption				
	Ever used	45	24 (53.3)	1.62 (0.87–3.01) Ref	0.124
	Never used	375	155 (41.3)		
13	Fruit intake				
	< 4 days/week	251	116 (46.2)	1.44 (0.97–2.15) Ref	0.069
	≥ 4 days/week	169	63 (37.3)		
14	Vegetable intake				
	< 4 days/week	17	10 (58.8)	1.97 (0.73–5.30) Ref	0.168
	≥ 4 days/week	403	169 (41.9)		
15	Physical activity (n = 116)				
	<600 MET min/week	44	22 (50.0)	1.48 (0.69–3.15) Ref	0.339
	≥600 MET min/week	72	29 (40.3)		
16	BMI				
	Underweight	8	3 (37.5)	0.78 (0.17–3.46) 0.70 (0.16–3.09) 0.87 (0.20–3.77) Ref	0.818
	Overweight	112	51 (45.9)		
	Obese	196	80 (40.6)		
	Normal	104	45 (43.3)		

*Statistically significant *p* < 0.05, MM, multimorbidity; APL, above poverty line; BPL, below poverty line; BMI, body mass index.

The multivariable logistic regression model showed that factors such as age, marital status, occupation, and tobacco use were the independent predictors associated with multimorbidity. Individuals aged 60 years and above had 3.03 (95% CI: 1.95–4.73) times higher odds of multimorbidity. Those who were unmarried/widowed/divorced had 2.15 (95% CI: 1.28–3.63) times more risk of multimorbidity compared to married individuals. Regarding employment status, those who were unemployed had 1.81 times higher risk of multimorbidity (95% CI: 1.14–2.87). In terms of tobacco use, individuals who had ever used tobacco had 3.72 times higher odds of multimorbidity compared to those who had never used tobacco (95% CI: 1.85–7.48; Table 3).

**Table 3:** Results of multivariable logistic regression analysis for independent predictors of multimorbidity.

Sl. No	Socio-demographic characteristics	Crude OR (with 95% CI)	Adjusted OR (with 95% CI)	*p*-value
1	Age			
	≥ 60 years	3.80 (2.50–5.77) Ref	3.03 (1.95–4.73) Ref	<0.001*
	<60 years			
2	Marital status			
	Others (unmarried, widowed, divorced)	2.66 (1.65–4.27) Ref	2.15 (1.28–3.63) Ref	0.004*
	Currently married			
3	Occupation			
	Unemployed	1.71 (1.12–2.61) Ref	1.81 (1.14–2.87) Ref	0.012*
	Currently employed			
4	Tobacco use			
	Ever used	3.28 (1.71–6.26) Ref	3.72 (1.85–7.48) Ref	< 0.001*
	Never used			

The symbol * in table indicates statistical significance, defined as *p* < 0.05.

Table 4 illustrates the results of the LCA model fit. The AIC and BIC values decreased from the one-class to the three-class models, and the G^2^ and χ^2^ values also showed a similar decline, reflecting an improvement in the model fit. However, starting with the four-class model, the AIC and BIC values began to rise, which means that adding more latent classes does not significantly improve the model. Based on these metrics—AIC, BIC, G^2^, and χ^2^—the 3-latent class model provided the best balance between goodness-of-fit and model simplicity.

**Table 4:** Model fit and diagnostic criteria for latent class analysis.

No: of latent classes	No: of estimated parameters	Residual degrees of freedom	Maximum log-likelihood	AIC	BIC	G^2^ (Deviance statistic)	$\chi^{2}$ (Chi-square goodness of fit)
1	12	408	−1410.546	2,845	2893.574	342.8207	40637.79
2	25	395	−1352.52	2755.045	2856.051	226.7738	1669.867
3	38	382	−1335.13	2746.253	2899.783	191.9826	471.8235
4	51	369	−1325.53	2753.066	2959.119	172.7958	447.9186
5	64	356	−1317.97	2763.93	3022.507	157.6596	310.7879

AIC, Akaike Information Criterion; BIC, Bayesian Information Criterion.

The results from class proportion and item-response probabilities (*ρ*) are presented in Table 5. The item responses with high probabilities were used to assign labels to the identified three-class model. Class 1 comprised the majority (73.42%) of the population and generally had low probabilities of having the majority of morbidities except for diabetes (0.40) and hypertension (0.23), making this a relatively healthy class. Class 2 represented 21.17% of the population and was labelled as cardiometabolic/mental and reported high probabilities of hypertension (1.00), diabetes (0.73), depression (0.40), and ischaemic heart disease (0.32), indicating a moderate risk class. Class 3 represented a small proportion of 5.41% of the study population was labelled as cardiorespiratory/metabolic/mental and reported high probabilities for a wide range of morbidities, including COPD/Asthma (1.00), hypertension (0.54), depression (0.53), ischaemic heart disease (0.50), and diabetes (0.48), making it the high-risk group. In all three latent classes, diabetes and hypertension emerged as common morbidities, indicating their widespread prevalence across different risk groups within the population.

**Table 5:** Class proportions and item-response probabilities from the three-latent class model of morbidities.

Latent class	1	2	3
Assigned labels	Relatively healthy	Cardiometabolic/mental	Cardiorespiratory/metabolic/mental
Class Proportion	73.42	21.17	5.41
Morbidities			
Diabetes	0.40	0.73	0.48
Hypertension	0.23	1.00	0.54
Ischaemic Heart Disease	0.05	0.32	0.50
Liver Disease	0.00	0.00	0.04
Arthritis	0.00	0.04	0.00
Thyroid Disease	0.09	0.24	0.26
Chronic Kidney Disease	0.00	0.01	0.13
Epilepsy	0.00	0.00	0.09
COPD/Asthma	0.03	0.00	1.00
Cancer	0.02	0.05	0.06
Stroke	0.00	0.11	0.13
Depression	0.11	0.40	0.53

Approximately a third of the households 32.4% (95% CI: 25.5–39.3) encountered catastrophic health expenditure. In this context, only a quarter of the individuals 24.6% (95% CI: 20.5–28.7) experiencing multimorbidity had some form of health insurance. The majority of this insurance 61.4% (95% CI: 56.7–66.0) was from the government.

Factors such as the age of the adult with multimorbidity (*p* < 0.05), socioeconomic status (p < 0.05), requirement of outpatient and inpatient services in the past month (*p* < 0.001), and mode of meeting expenses during the healthcare visit in the previous month (*p* < 0.001) had a significant association with catastrophic health expenditure on the bivariate analysis (Table 6). Households with individuals aged 60 years and above who have multimorbidity were 2.39 (95% CI: 1.99–5.77) times and utilisation of outpatient services in the past month were found to have 4.09 (95% CI: 2.01–8.32%) times more risk of incurring catastrophic health expenditure compared to their counterparts (Table 7).

**Table 6:** Catastrophic health expenditure and its determinants.

Sl. No	Variable	Total (n)	CHE present n (%)	Crude OR (with 95% CI)	*p*-value
1	Location of household				
	Rural	105	33 (31.4)	0.89 (0.47–1.69) Ref	0.740
	Urban	74	25 (33.8)		
2	Type of family				
	Nuclear	74	25 (33.8)	1.11 (0.59–2.09) Ref	0.740
	others	105	33 (31.4)		
3	Socioeconomic status				
	APL	109	42 (38.5)	2.11 (1.07–4.16) Ref	0.029*
	BPL	70	16 (22.9)		
4	Age of adult with multimorbidity				
	≥ 60 years	130	50 (38.5)	3.20 (1.38–7.38) Ref	0.006*
	<60 years	49	8 (16.3)		
5	Educational status				
	Below high school	73	25 (34.2)	1.15 (0.61–2.17) Ref	0.662
	High school and above	106	33 (31.1)		
6	Occupational status				
	Homemaker	99	26 (26.3)	1.81 (0.90–3.65) 0.90 (0.34–2.39)	0.149
	Employed	24	10 (41.7)		
	Unemployed	56	22 (39.3)		
7	Marital status				
	Currently married	122	42 (34.4)	1.34 (0.67–2.67) Ref	0.397
	Others (unmarried, divorced, widowed)	57	16 (28.1)		
8	Family size				
	≤ 4	94	33 (35.1)	1.29 (0.69–2.43) Ref	0.416
	>4	85	25 (29.4)		
9	Outpatient services in the past month				
	Yes	89	43 (48.3)	4.67 (2.33–9.34) Ref	<0.001*
	No	90	15 (16.7)		
10	In-patient services in the past month				
	Yes	11	11 (100.0)		<0.001*^#^
	No	168	47 (28.0)		
11	Mode of meeting healthcare expenses in the past month				
	Government subsidised	42	0 (0)		<0.001*^#^
	Out of pocket	128	53 (41.4)		
	Health insurance	9	5 (55.6)		
12	Health insurance coverage				
	Not insured	135	45 (33.3)	1.19 (0.56–2.49) Ref	0.641
	Insured	44	13 (29.5)		
13	Type of insurance				
	Government	27	6 (22.2)	0.40 (0.10–1.53) Ref	0.180
	Private	17	7 (41.2)		

*Statistically significant *p* < 0.05, # Fisher’s exact test, CHE, catastrophic health expenditure; APL, above poverty line; BPL, below poverty line.

**Table 7:** Multivariable logistic regression analysis for identifying the independent predictors of catastrophic health expenditure among households.

Sl. No	Variable	Crude OR (with 95% CI)	Adjusted OR (with 95% CI)	*p*-value
1	Age of adult with multimorbidity			
	≥ 60 years	3.20 (1.38–7.38) Ref	2.39 (1.99–5.77) Ref	0.049*
	>60 years			
2	Outpatient services in the past month			
	Yes	4.67 (2.33–9.34) Ref	4.09 (2.01–8.32) Ref	<0.001*
	No			
3	Socioeconomic status			
	APL	2.11 (1.07–4.16) Ref	1.91 (0.93–3.96) Ref	0.078
	BPL			

*Statistically significant *p* < 0.05, APL, above poverty line; BPL, below poverty line.

The distribution of Out-of-Pocket Expenditure (OOPE) among the different multimorbidity groups is shown in Table 8. The median OOPE was found to be higher among the penta and above multimorbidity group than the other groups (dyad, triad, and quad). Multiple linear regression analysis to explore potential factors influencing OOPE observed that inpatient (IP) services and the total number of morbidities were significant predictors of healthcare costs. IP services emerged as the stronger predictor (*β* = 0.557, *p* < 0.001), with patients who utilised IP services incurring an average additional cost of ₹22,082.37 (95% CI: ₹17,139.88 to ₹27,024.86) compared to those who did not. Additionally, each extra morbidity (from dyad to penta or more) was associated with an increase in healthcare costs by ₹1,278.75 (*β* = 0.128, *p* = 0.044, 95% CI: ₹35.58 to ₹2,521.92; Table 9).

**Table 8:** Distribution of out-of-pocket expenditure (OOPE) across multimorbidity groups.

Group	Minimum	25th Percentile (Q1)	Median	75th Percentile (Q3)	Maximum
Dyad	0	400.00	891.000	2477.750	84432.000
Triad	0	96.250	950.000	2061.250	50000.000
Quad	0	76.250	727.500	3622.500	60000.000
Penta and above	0	555.000	1177.000	3508.000	30675.000

**Table 9:** Multiple linear regression analysis for identifying the independent predictors of out-of-pocket health expenditure.

Predictor	B (Unstandardized coefficient)	Std. error	*t*	95% Confidence interval for B		*p*-value
				Lower bound	Upper bound	
IP services	22082.37	2504.386	8.817	17139.88	27024.86	<0.001
Total morbidities	1278.752	629.922	2.03	35.58	2521.924	0.044

## 4 Discussion

In the present study, non-communicable disease multimorbidity was prevalent in 42.6% of the individuals aged 40 years and above in Ernakulam district. Older adults, as well as those who were unmarried/widowed/divorced, unemployed, and individuals with a history of tobacco use, were more likely to have multimorbidity. Our study provides initial estimates of the costs related to multimorbidity. Approximately a third of the households 32.4% encountered catastrophic health expenditure. Households with individuals aged 60 years and above having multimorbidity, the utilisation of outpatient services in the past month had a double and quadruple times higher risk of incurring catastrophic health expenditure than their counterparts.

The higher prevalence of multimorbidity in Kerala may be due to the advanced stage of epidemiological and demographic transition. The state’s rapid urbanisation, changing lifestyles, and ageing population have contributed to a rising burden of chronic conditions. Kerala’s high literacy rates and healthcare awareness create both opportunities and challenges in dealing with multimorbidity (25).

Studies from various parts of Kerala, hospital-based and community-based studies have also reported a similar prevalence ranging from 39.8% (10) to 45.4% (8), respectively. However, another study in six states of India (26) among younger individuals aged 18 years and above, reported a lower prevalence of 8.9%. Secondary data analysis from a national survey revealed the multimorbidity prevalence to be 30.7% in the older adults aged 60 years and above (7). In a systematic review and meta-analysis conducted across 54 countries, multimorbidity was 37.2% (27).

An increase in age above 60 years has been found to elevate the risk of multimorbidity, a finding corroborated by both Indian and global studies (3, 4, 28). The ageing population is more prone to multimorbidity due to several factors related to ageing, such as prolonged exposure to risk factors, natural physiological decline, cascading health issues where one condition triggers others, increased use of medications, and the associated side effects. A recent systematic analysis (27) has also found the same. In Kerala, where life expectancy is higher than the national average, the extended lifespan provides more time for multiple chronic conditions to develop and coexist.

In the present study, individuals who were unmarried, widowed, or divorced also had a high risk of multimorbidity compared to those who were married. A longitudinal study in multiple countries showed that those who were widowed, divorced, or separated had higher odds of multimorbidity (29). Unmarried/widowed/divorced individuals might face barriers in accessing healthcare services and seeking healthy behaviours due to financial constraints, and lack of social and family support, leading to less healthy lifestyle choices and an increased risk of developing multiple chronic conditions (30).

In our study, it was observed that unemployed individuals had an increased risk of multimorbidity. The finding was consistent with a previous study in Kerala, where unemployed individuals had higher odds of multimorbidity (8). The higher prevalence of multimorbidity in unemployed individuals could potentially be due to inadequate physical activity and unhealthy dietary habits. Our study also found a higher odds of multimorbidity among those who ever used tobacco. Increased risk of multimorbidity among smokers was also confirmed by an English longitudinal study of ageing (31).

In this study, dyads (comprising two chronic conditions) were the most prevalent pattern of multimorbidity, affecting 21.9% of participants, followed by triads at 13.3%. This pattern appears to be consistent globally, with similar findings reported in countries such as India, China (32, 33), and various developed nations. This was further underscored by the latent class analysis where diabetes and hypertension (metabolic cluster) were found to have high probabilities across all three latent classes. As highly prevalent conditions in all multimorbid classes, diabetes and hypertension may serve as gateway diseases, facilitating the development of other non-communicable diseases.

Our study found that approximately a third 32.4% households of adults living with multimorbidity incurred CHE and this was more among households with older adult members. Outpatient care in the preceding month also led the patient’s family to incur catastrophic health expenditures. The National Sample Survey Office (NSSO) 75th round (2017–2018) reported that approximately 63.3% of households incurred catastrophic spending (using a 10% threshold) under an NCD multimorbidity scenario. They also noted that outpatient care for conditions such as cancer and cardiovascular diseases is underfinanced by 45–50% in the presence of multimorbidity in comparison to standalone diseases (16). CHE prevalence was, however, lower than 45.6% in a longitudinal study conducted in households of older adults (≥60 years) living with multimorbidity in China (34). Older adults with chronic diseases or multimorbidity often require long-term medication and regular medical check-ups, leading to increased demand for medical services and imposing economic burdens on their families. Despite the importance of health insurance in preventing catastrophic health expenditure, our study did not find any significant differences in its protective role compared to households without insurance.

### 4.1 Strengths

With Kerala bearing a significant burden of NCDs, this study, with a representative sample of 420 participants from both rural and urban areas, offers valuable insights into the current prevalence and risk factors of multimorbidity. To our knowledge, this is the first-ever study conducted in Kerala to estimate the catastrophic health expenditure encountered by households while treating adults with multimorbidity. The findings of this study could pave the way for further research initiatives within the state and can also guide the policymakers to formulate strategies ensuring financial risk protection among households in the future.

### 4.2 Limitations

There is a possibility of recall bias since multimorbidity was determined through self-reporting, which may lead to an underestimation of its true prevalence. However, the high literacy rate, strong health awareness, and proactive healthcare-seeking behaviour of the population reduce the likelihood of underestimating or overestimating the issue. In this study, a 1-month recall was used to estimate the health expenditure among the households, which might influence the accuracy of the results. CHE was estimated based on the previous month. This may not be representative of the year.

## 5 Conclusion

The research underscores the increasing burden of multimorbidity among the older adults, unmarried individuals, unemployed, and tobacco users, emphasising the urgent need for the National Programme for the Prevention and Control of Non-Communicable Diseases to formulate comprehensive protocols that address multimorbidity among these groups. These protocols should be facilitated through comprehensive health assessments and integrated management strategies for patients with multiple co-existing conditions. To protect households from significant catastrophic health expenditures, the research highlights the necessity for drafting better policies that provide financial protection to households in the future.

## Data Availability

The raw data supporting the conclusions of this article will be made available by the authors, without undue reservation.
